# Supplementary material for: Identification of photoperiod-induced specific miRNAs in the adrenal glands of Sunite sheep (Ovis aries)
Source: Front Vet Sci. 2022 Jul 22;9:888207. doi: 10.3389/fvets.2022.888207 (PMC9354845; doi:10.3389/fvets.2022.888207)
Supplement: Supplementary file 1 [file Data_Sheet_1.ZIP › Supplementary file/miRDeep2 file.zip!/miRDeep2 file/Know_Structure/LP42A2/oar-mir-758.pdf]

miRBase precursor : oar-mir-758
Total read count : 431
oar-miR-758-5p read count : 0
oar-miR-758-3p read count : 430
remaining reads : 1

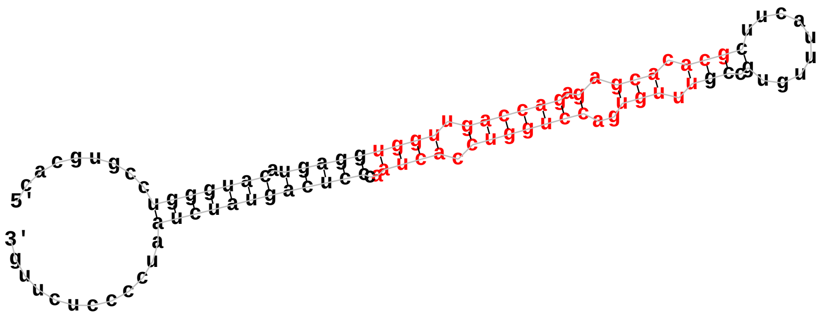

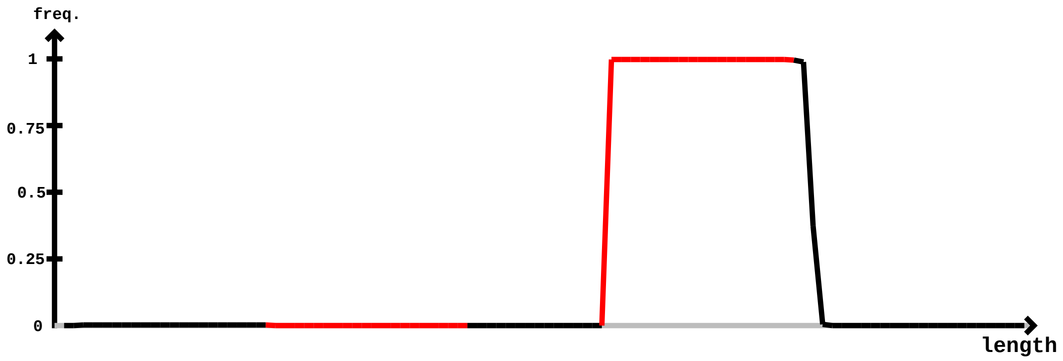

oar-miR-758-3p
oar-miR-758-5p

```
5'- cacgugccuggguacaugaggugguugaccagagagcacacgcuucauuugugccguuugugaccugguccacuaacccucaguaucuaaucccccucuug   -3'    exp
    ........(((((((.(((((((((.((((((.(.(((.((((.........).))).)))..))))))).)))).)).))))))))))............    reads      mm         sample
    .acgugccuggguacaugagg................................................................................    1          0          seq
    ........................................................uuugugaccugguccacua..........................    1          0          seq
    ........................................................uuugugaccugguccacuaa.........................    3          0          seq
    ........................................................uuugugaccugguccacuaac........................    265        0          seq
    ........................................................uuugugaccugguccacuaacc.......................    159        0          seq
    ........................................................uuugugaccugguccacuaaccc......................    2          0          seq
```
